# Prevalence and risk factors associated with tropical theileriosis in Egyptian dairy cattle

**DOI:** 10.14202/vetworld.2022.919-924

**Published:** 2022-04-14

**Authors:** Abdelfattah Selim, William Weir, Hanem Khater

**Affiliations:** 1 Department of Animal Medicine (Infectious Diseases), Faculty of Veterinary Medicine, Benha University, Toukh 13736, Egypt; 2 Institute of Biodiversity Animal Health and Comparative Medicine, School of Veterinary Medicine, College of Medical, Veterinary and Life Sciences, University of Glasgow, 464 Bearsden Road, Glasgow, G61 1QH, UK; 3 Department of Parasitology, Faculty of Veterinary Medicine, Benha University, Toukh 13736, Egypt

**Keywords:** cattle, Egypt, polymerase chain reaction, risk factors, *Theileria annulata*

## Abstract

**Background and Aim:**

*Theileria annulata* is the most prevalent piroplasm infecting cattle across Egypt. Theileriosis is transmitted by ixodid ticks of the genus *Hyalomma*. This study aimed to estimate the prevalence of theileriosis in cattle and their associated risk factors for infection.

**Materials and Methods:**

A total of 570 blood samples were collected from cattle from five governorates (administrative districts) in Egypt and examined using a polymerase chain reaction assay to estimate the prevalence of tropical theileriosis and assess the associated risk factors

**Results:**

The overall prevalence rate was 16.49%, with the Alexandria district having the highest prevalence. The results revealed that the risk of theileriosis was elevated in older cattle (odds ratio [OR]=8.9, 95% confidence interval [CI]: 3.6-21.9), especially in summers (OR=3.07, 95% CI: 1.4-6.3). Cattle heavily infested with ticks were at particular risk (OR=3.05, 95% CI: 2.1-4.5), as were those to which acaricide had not been applied (OR=13.7, 95% CI: 5.6-33.6).

**Conclusion:**

Understanding the risk factors associated with *T. annulata* infection and regular infection monitoring could reduce infection rates and economic losses and is essential for the implementation of efficient control programs.

## Introduction

Ticks are obligate hematophagous ectoparasites of humans and animals. They transmit more species of pathogens than any other blood-feeding arthropod [1-6] and require careful monitoring and control measures [7,8]. A variety of tick-borne diseases affect animal productivity in developing countries [9]. Bovine tropical theileriosis is a tick-borne protozoal disease caused by *Theileria annulata* and is transmitted by ixodid ticks of the genus *Hyalomma* [10,11]. Theileriosis causes large financial losses to farmers because of debility, direct death, morbidity, milk loss, and control costs for acaricides, treatment, and vaccines [12]. Although *T. annulata* cause larger productivity losses in exotic cattle breeds and their crosses, naive indigenous cattle, particularly calves and adults under endemic instability, are also affected [13]. Furthermore, farmers’ coping strategies for acaricide failure, such as raising acaricide concentration and application frequency or admixing acaricides, accelerate resistance, deplete earnings, and exacerbate poverty [14].

The disease occurs in many parts of the world, including South Europe, Asia, and North Africa [15,16]. The disease is characterized clinically by enlarged lymph nodes during the lymphoproliferative phase, followed by pyrexia, anemia, and associated leukopenia during the lymphodestructive phase.

*T. annulata* has been reported in different locations in Egypt [17]. It can be detected using various diagnostic methods [18,19]. A direct microscopic smear is a rapid and cheap technique, but it provides low sensitivity and is unsuitable for epidemiological studies [20]. By contrast, polymerase chain reaction (PCR)-based assays used to detect parasite DNA are characterized by high sensitivity and may be used to unequivocally identify the species of piroplasm present [21]. Only a few studies have investigated the epidemiology of tick-borne diseases in Egypt. The previously reported prevalence of *T. annulata* was 16.05% in the Menofia [22] and 11.31% in the Gharbia [23] districts in North Egypt, and it was 11.1% in Qena [24] in South Egypt. Nevertheless, these studies have focused on a restricted number of locations [25,26].

Consequently, the study aimed to estimate the molecular prevalence of *T. annulata* among cattle across North Egypt and assess risk factors associated with its infection across the region.

## Materials and Methods

### Ethical approval

The Internal Ethics Review Committee of the Faculty of Veterinary Medicine, Benha University, approved (Approval Number: BUFVTM) this study. All sample collection activities followed ethical guidelines.

### Study period and location

**Figure-1:**
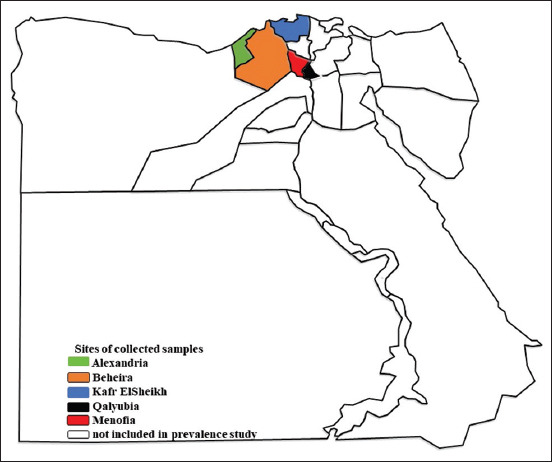
Map showing the location of the study areas [Map generated by QGIS software].

An epidemiological study was conducted from April 2019 to March 2020 in five North Egyptian districts, primarily Alexandria, Beheira, Kafr El Sheikh, Qalyubia, and Menofia (Figure-1). These represent agricultural regions with a high density of cattle used for milk and beef production. All the selected governorates (districts) are situated in the Nile Delta region except Alexandria, which is located on the Mediterranean Sea. The Delta region has a hot desert climate (Köppen: BWh) like the rest of Egypt. The hottest months in the Delta region are July and August, with an average high temperature of 34°C. Winter temperatures typically vary from 9°C at night to 19°C during the day. The Nile Delta region gets quite humid during the winter months because of colder temperatures and some rain.

### Sample size estimation

The sample size for the study was determined using Cochran’s formula [27] as follows:


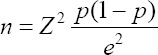


Where, n is the sample size, *Z* is the level of confidence required (95% confidence interval [CI]), p is the expected prevalence (10.25) % for *T. annulata*, as previously reported in Egypt [17], and e is the required level of precision 5% based on Pourhoseingholi *et al*. [28].

### Sample collection

A total of 570 blood samples were collected from dairy cattle representing the five selected governorates (districts) in Egypt. The sample collection represents both sexes (180 males and 390 females) during four seasons (spring, summer, autumn, and winter), several different breeds (93 Baladi, 210 Friesian, and 267 mixed), and three age groups (<2, 2-4, and >4 years). In addition, managemental factors such as tick infestation and monthly application of acaricides were determined. Blood samples (5 mL) were collected from the jugular vein using a vacuum tube with ethylenediaminetetraacetic acid and transported in an icebox (4°C) to a Veterinary Diagnostic Laboratory, Benha University, and preserved at −20°C for molecular analysis.

### Molecular analysis

Total genomic DNA was extracted from all blood samples using a QIAamp® DNA Mini (Qiagen GmbH, Hilden, Germany) kit following the manufacturer’s instructions. Extracted DNA was kept at −20°C.

The PCR assay was performed using specific primers targeting *T. annulata* *Tams*-1 gene, which was evaluated by D’Oliveira *et al*. [29]. The forward primer (5′-GTAACCTTTAAAAACGT-3′) and reverse primer (5′-GTAACCTTTAAAAACGT-3′) were used. The PCR reaction was performed in a total volume of 25 μL, containing 12.5 μL Dream Taq Green PCR master mix (2×) (Thermo Scientific, Germany), 1 μL of each primer (20 pmol/μL), 5.5 μL nuclease-free water, and 5 μL DNA template. The PCR reaction was conducted at 94°C for 3 min followed by 35 amplification cycles. Each cycle includes a denaturation step at 95°C for 20 s, an annealing step at 56°C for 45 s, and an extension step at 72°C for 50 s. There was also a final extension for 10 min at 72°C. The PCR products were separated and visualized through electrophoresis (Cleaver Scientific Ltd, UK) on a 1.5% agarose gel.

### DNA sequencing

Two PCR products were randomly selected, purified, and cleaned for sequencing. Direct sequencing was performed in both directions utilizing the same pair of primers as the PCR reaction using the ABI PRISM^®^ BigDye™ Terminators v3.1 Cycle Sequencing Kit (Applied Biosystems, USA). The obtained sequences were trimmed using the BioEdit [30] program and were compared with the NCBI non-redundant database using the BLAST (https://blast.ncbi.nlm.nih.gov) search tool. The sequences of two amplicons were confirmed and represented *T. annulata*. They were deposited in GenBank under accession numbers LC549653 and LC549654.

### Statistical analysis

The data were analyzed by statistical package for the social sciences v 17.0 (IBM Corp., NY, USA) using the Chi-square test. Differences were considered significant if p*<*0.05. Logistic regression analysis was performed to assess the effect of each variable on the prevalence of *T. annulata*. The association between *T. annulata* infection and factors was investigated using univariable analysis. Risk factors, odds ratio (OR), and CIs for each significant variable (p<0.2) were identified using a multivariable logistic regression model.

## Results

### Prevalence of T. annulata according to the locality

Overall, the prevalence rate of *T. annulata* among cattle in the studied areas was 16.49%. The Alexandria governorate had the highest prevalence rate (22.58%) compared with Menofia, Beheira, Kafr El Sheikh, and Qalyubia (14.62%, 13.91%, 12.22%, and 16.25%, respectively) (Table-1). There was no significant difference in the prevalence of *T. annulata* among the different localities (p=0.1).

**Table 1:** Prevalence rate of *Theileria annulata* in cattle in examined areas.

Locality	Number of examined animals	Number of positive animals	%	95% CI	p-value
Alexandria	155	35	22.58	16.4-30.1	0.1**
Beheira	130	19	14.62	9.2-22.1	
Kafr El Sheikh	115	16	13.91	8.4-21.9	
Qalyubia	90	11	12.22	6.5-21.2	
Menofia	80	13	16.25	9.2-26.5	
Total	570	94	16.49	13.5-19.8	

** The results are not significant at p*<*0.05. CI=Confidence interval

### Univariate analysis of risk factors associated with *T. annulata* infection

Univariate logistic regression analysis was performed for six variables to assess the risk factors that could affect *T. annulata* prevalence (Table-2). The results showed that the prevalence of theileriosis increased significantly (p=0.0001) with the age of the examined animals, reaching 31.8% in older cattle (>4 years old). In contrast, it was 3.5% in younger animals (<2 years old) and 16.8% in the middle age group (2-4 years old).

**Table 2:** Univariate analysis of associated risk factors for *Theileria annulata* infection.

Parameter	Number of examined animals	Number of positive	%	95% CI	p-value
Age				
<2	140	5	3.5	1.3-8.5	0.0001*
2-4	320	54	16.8	13-21.5	
>4	110	35	31.8	23.4-41.4	
Sex				
Male	180	25	13.9	9.3-20	0.2
Female	390	69	17.7	14.1-22	
Breed				
Baladi	93	15	16.1	9.6-25.5	0.5
Friesian	210	39	18.6	13.6-24.6	
Mixed	267	40	15	11-20	
Season				
Spring	120	8	6.6	3.1-13.1	0.0001*
Summer	205	43	21	15.7-27.3	
Autumn	145	35	24.1	17.6-32	
Winter	100	8	8	3.7-15.6	
Tick infestation				
Yes	215	61	28.4	22.5-34.9	0.0001*
No	355	33	9.2	6.5-12.9	
Acaricide application				
Regular	130	5	3.8	1.4-9.2	0.0001*
Irregular	387	61	15.7	12.3-19.8	
Not in use	53	28	52.8	38.7-66.4	

95% CI=95% confidence interval.

* The result is significant at p*<*0.05

The prevalence of *T. annulata* showed a significant disparity between different seasons (p=0.0001), with higher infection rates in autumn (24.1%) and summer (21%) compared with winter (8.0%) and spring (6.6%). Infection prevalence is strongly associated with three other factors: The degree of tick infestation, the frequency of acaricidal application, and the animal’s age. Tick-infested cattle displayed a higher prevalence of *T. annulata* than uninfected animals (28.4% vs. 9.2%, p=0.00001). Cattle not treated with acaricides showed a far higher infection prevalence than those treated regularly (52.8% vs. 3.8%, p=0.00001). The sex and breed of animals did not significantly affect the prevalence of *T. annulata* infection (Table-2).

### Multivariate logistic regression analysis

Based on the results of the univariate testing, four risk factors were selected for multivariate analysis. The significance and OR were calculated for animal age, season sampled, level of tick infestation, and acaricidal application frequency. Cattle over 4 years old were found to be 8.9 times more likely to be positive than those below 2 years old. Furthermore, the prevalence of *T. annulata* was 4 times higher in autumn and 3 times higher in summer than that in spring. The multivariate analysis indicated that infection prevalence was 3 times higher among cattle that exhibited heavy tick infestation than uninfested animals. Similarly, cattle exposed to acaricides are 14 times less likely to be infested with ticks than those in which acaricides were not used (Table-3).

**Table 3:** OR from logistic regression analysis of potential risk factors associated with *Theileria annulata* infection.

Risk factor	Comparative parameter	OR	95% CI
Age	<2	ref	
	2-4	4.7	1.9-11.5
	>4	8.9	3.6-21.9
Season	Spring	ref	
	Summer	3.07	1.4-6.3
	Autumn	3.6	1.7-7.5
	Winter	1.2	0.46-3.08
Tick infestation	No	ref	2.1-4.5
	Yes	3.05	
Acaricide application	Regular	ref	
	Irregular	4.09	1.6-9.9
	Not in use	13.7	5.6-33.6

95% CI=95% confidence interval, OR=Odds ratio

## Discussion

Tropical theileriosis is an endemic disease of cattle in Egypt and is considered an impediment to livestock production due to the severe economic losses it causes [26]. This study investigated the prevalence of *T. annulata* infection in the Nile Delta and assessed the risk factors associated with infection in this region.

Tropical theileriosis has been previously reported in different localities of Egypt, with a prevalence rate of 11.6% in North Egypt [17]. In the present study, the overall prevalence across the five study areas was 16.5%. The highest rate was observed in Alexandria (22.5%) and Menofia (16.2%). These data for Menofia are in line with the previously reported rate of 16.05% [22]. However, in every area, the prevalence was lower than the rate of 63.6% reported in the El-Wady El-Gaded governorate in South Egypt [26]. The high prevalence could be attributed to the lower cattle population, relatively higher temperatures year-round, favoring tick prevalence, poor veterinary observation or absence of veterinary care, and regular application of acaricides. The Mediterranean climate of the present study is characterized by rainfall in the winter, moderate rain in autumn, and high temperature in summer. Such seasonal fluctuations provide a suitable environment for tick propagation, which is the sole vector of theileriosis [31-33].

The overall prevalence of *T. annulata* in this study (18.33%) was lower than that reported in Pakistan (29.9%) [9] and Sudan (39%) [34]. The variation in the prevalence of theileriosis between countries may be attributed to geographical or ecological factors, animal breed, management practice, and tick control [35-37].

The present study revealed that the prevalence of theileriosis was higher in older (>4 years old) and middle-aged (2-4 years old) cattle compared with younger animals (<2 years old). These results agree with the previous findings in Egypt, where younger animals showed a lower prevalence of infection than adults [34,38]. Because of the long-term nature of infection with *T. annulata*, the higher prevalence in older animals may reflect increased exposure to infection or management-associated factors. In contrast to babesiosis, where inverse age immunity has been documented, this phenomenon has not been established for tropical theileriosis.

Despite the non-significant difference in the effect of sex and breed of examined animals on the prevalence of theileriosis, the prevalence rate was higher in female cattle, as shown in a previous study [10,36]. Such findings could be related to stress factors such as pregnancy, parturition, and milk production [26]. This study revealed the low prevalence rate among Friesian cattle compared with the other breeds. This finding may be associated with a high level of nutrition and regular application of acaricides, which will reduce cattle exposure to tropical theileriosis [39,40].

This study indicated that no or irregular acaricide application is constant with higher *T. annulata* infection rates and vice versa. Similar findings were reported by Miyama *et al*. [41] and Moumouni *et al*. [42]. Such observation could be explained as ticks are the sole vector of theileriosis and highlight the role of regular acaricide application, which is one of the main strategies for preventing the disease in a herd.

The present study’s seasonal finding indicated that the highest prevalence rates occurred in autumn and summer, which is in consistent with previous findings [9,34]. The higher prevalence in the dry season may be due to poor pasture conditions and inadequate nutrition, resulting in a weakened immune system [43]. This evidence may be attributed to the higher number of examined cattle in the summer season. Furthermore, because ixodid ticks are the principal vectors of *T. annulata* infection transmission, the high incidence of ixodid tick species during the summer season increases the likelihood of infection with *T. annulata*.

## Conclusion

An appreciable prevalence of *T. annulata* infection was recorded in all Nile Delta areas given that tropical theileriosis is known to be endemic across such regions. Risk factor analysis confirmed that the tick infestation level is critical for the prevalence of infection, especially among older cattle in autumn and summer. Thus, regular monitoring of *T. annulata* infection and tick control programs should be implemented to decrease the infection rate and economic losses.

## Authors’ Contributions

AS, WW, and HK: Conceptualization, methodology, formal analysis, investigation, resources, data curation, and writing – original draft preparation. AS and HK: Writing – review and editing. AS and HK: Project administration. AS, WW, and HK: Funding acquisition. All authors have read and approved the final manuscript.
